# Person-centered shared decision-making in district nursing care on interventions to support independence in older adults with multiple chronic conditions: a video observation study

**DOI:** 10.1186/s12912-025-03778-3

**Published:** 2025-09-26

**Authors:** Sigrid Wulfse-Huisman, Dorien Oostra, Jessica Veldhuizen, Nienke Bleijenberg, Bianca Buurman-van Es, Ruth Pel-Littel

**Affiliations:** 1 Department of Internal Medicine, Section of Geriatrics and Amsterdam Public Health, Academic Medical Center, University of Amsterdam, Meibergdreef 9, Amsterdam, 1105 AZ The Netherlands; 2 Amstelring District Nurse Care, Laan van de Helende Meesters 431, Amstelveen, 1186 DK The Netherlands; 3 Department of Health and Vitality, HAN University of Applied Sciences, Kapittelweg 33, Nijmegen, 6525 EN The Netherlands; 4 Research Centre for Healthy and Sustainable Living, Faculty of Health Care, University of Applied Sciences Utrecht, Postbus 12011, Utrecht, 3501 AA The Netherlands; 5 The Dutch Nursing Society, Orteliuslaan 1000, Utrecht, 3528 BD The Netherlands; 6 Department of General Practice, Division Julius Center for Health Sciences and Primary Care, University Medical Center Utrecht, Universiteitsweg 100, Utrecht, 3584 CG The Netherlands; 7 Vilans, Center of Expertise for Long-term Care, Postbus 8228, Utrecht, 3503 RE The Netherlands

**Keywords:** Home-dwelling older adults, Multiple chronic conditions, Supporting independent functioning, Person-centered shared decision-making, District nursing care, Home care

## Abstract

**Background:**

Older adults with multiple chronic conditions often have care needs who are complex, diverse, and intertwined with personal values and goals for independent functioning. Healthcare professionals achieve person-centered decision-making by aligning interventions with prioritized personal and care-related goals. Within the context of district nursing care (home-based nursing care), person-centered shared decision-making plays a critical role in supporting older adults to maintain their independence. However, there is limited insight into how person-centered shared decision-making about interventions to support independence is applied in district nursing practice.

**Objective:**

This study explored how district nurses engage in person-centered shared decision-making with older adults with multiple chronic conditions during discussions about interventions to support independent functioning at home. It examined how verbal and non-verbal communication contributes to the decision-making process and how these practices align with existing person-centered shared decision-making models.

**Methods:**

A non-participatory video observation design was used. Sixteen video recordings of intakes and care evaluations were collected across four district nursing organizations in the Netherlands. Verbal communication was reported using the Consolidated Criteria for Reporting Qualitative Research checklist, while non-verbal communication was analyzed using the guideline of Systematic Coding of Observed human Behaviour.

**Results:**

Analysis of verbal and non-verbal communication showed that district nurses appeared friendly, interested, and encouraged older adults to speak. In addition, verbal communication analysis revealed that independent functioning was not assessed or decided upon in a structured, goal-oriented way. Independence was mainly discussed when raised by older adults or related to the referrer’s care request. Eight verbal communication themes emerged: being informed at start of the conversation; initiating the conversation; exploring care needs; demonstrating attentiveness to the older adult; identifying care problems and care goals; presenting and discussing options; agreeing on district nursing care, and concluding the conversation.

**Conclusion:**

A person-centered shared decision-making model is crucial in district nursing care to support informed and collaborative decisions about interventions that promote independent functioning in older adults with multiple chronic conditions. This model should include preparatory and introductory phases, emphasize trust-building, and be co-developed with both district nurses and older adults. Effective implementation requires integration into professional education and training, as well as the development of a holistic geriatric assessment framework and communication skills training tailored to the context of district nursing care.

**Trial registration:**

Not applicable for this study.

**Supplementary Information:**

The online version contains supplementary material available at 10.1186/s12912-025-03778-3.

## Background

Shared decision-making (SDM) is essential for older adults with more than two chronic conditions, referred to as multiple chronic conditions (MCC) [1]. Their care needs are often complex, diverse, and rooted in interconnected goals related to symptoms, daily functioning, and personal values [2]. Shared decision-making aims to discuss a stratification of these goals with older adults with MCC, informal caregivers and formal caregivers, before a decision is made [2]. In this study, the approach to shared decision making (SDM) was aligned with Elwyn et al. [2]. In this approach, healthcare professionals achieve person-centered decision-making by eliciting and prioritizing personal and care-related goals and aligning prioritized goals and interventions.

“The Dynamic Model of Shared Decision-Making in frail older patients” [3] and “The Model for Goal-Based Shared Decision-Making” [2] provide structured approaches to guide person-centered SDM in this population. These models emphasize goal setting, exploration of options, and collaborative decision-making as a dynamic and iterative process involving older adults, their informal caregivers, and health professionals. They have been tested and applied in clinical settings, including hospitals and general practitioner practices in the Netherlands, and have shown promise in improving person-centered care [4]. Additionally, a preparatory SDM tool has been developed to help older adults describe and prioritize their care needs and goals, supporting meaningful engagement in the decision-making process [5]. Implementation of these models necessitates effective verbal and non-verbal communication by healthcare professionals [15–17]. Older adults value healthcare professionals who listen [18], communicate clearly [19], show empathy and interest [20], and avoid rushing decisions [19]. Older adults also appreciate being invited into the SDM process, given time to prepare [21], and having roles within SDM clearly explained [22, 23]. Verbal communication, such as providing information, and non-verbal cues, like affirmative nodding, build trust and engagement in care decisions [24].

Since older adults with MCC have many diverse care needs [1, 6], they often receive nursing care at home [7]. In light of the global demographic shift toward an ageing population and the growing emphasis on enabling older adults to age in place, the role of home care nurses has become increasingly vital [8]. Across health systems worldwide, home care nurses are at the forefront of facilitating person-centered SDM with older adults with MCC, particularly in making tailored decisions about interventions that support independent functioning at home [9–11], particularly during intake and evaluation conversations when care plans are developed in collaboration with older adults [12]. In the Netherlands, nursing care at home is referred to as district nursing care, which is grounded in the concept of Positive Health—a holistic, person-centered vision that focuses on individuals’ ability to adapt and self-manage across physical, emotional, and social domains [13]. A key goal of Dutch district nursing care is to preserve and improve the independent functioning of older adults living at home [14]. Compliant with Elwyn et al. [2], Positive Health [13] and The Health Council of the Netherlands [15], we composed the following description of person-centered SDM with older adults in district nursing care, on interventions to support independent functioning: “Setting goals, discussing options and preferences and deciding collaboratively on interventions that match older adults’ perceived health and support them to function in their physical, social and emotional life domains and to manage these domains to enable them to continue to live safely and independently at home for as long as possible, with or without (in)formal care”.

While models and communication skills for person-centered SDM are recognized as important, they may not be sufficiently embedded in district nurses’ education and daily practice [16, 17]. Despite the central role of district nurses in SDM with older adults on interventions to support physical, social, and emotional independent functioning [18, 19], research has also shown that little is known about how person-centered SDM unfolds in real-life district nursing care interactions, especially about how verbal and non-verbal communication supports this process [20].

Understanding of the current practice of SDM in district nursing care on interventions to support independence in older adults may provide information for optimizing person-centered district nursing care for older adults with MCC [21]. Subsequently, it could help maintain and improve the independence of older adults, allowing them to continue to live at home as they choose, despite their MCC. Therefore, this study explored how district nurses use verbal and non-verbal communication during decision-making about interventions to support independent functioning in older adults with MCC, and how their shared decision-making aligns with existing person-centered SDM models.

## Method

### Design and setting

This study was created through a non-participating video observation design. Observations were used to understand SDM of district nurses, while the researcher remained separate from the observed activities [22]. It is part of the Data Nurse project in the Netherlands: “Data Driven Essential Care in District Nursing Care: improving patient outcomes and maintaining independence”. In this project patients and district nursing teams of five organizations jointly aim to increase independent functioning in older adults with district nursing care. Four Data Nurse-affiliated organizations participated in the observations. The observed district nurses were employed by one of these four organizations. For transparent reporting of the verbal communication, the Consolidated Criteria for Reporting Qualitative Research (COREQ) checklist was used [23]. To interpret, quantify, and analyse non-verbal communication, the guideline of Systematic Coding of Observed human Behaviour (SCOBe) was used [24].

### District nursing care in the Netherlands

We define district nursing care as a holistic approach encompassing preventive, supportive, and rehabilitative care, including technical, psychosocial, and personal services nurses provide to individuals and communities [25]. More than half a million people in the Netherlands receive district nursing care, and 80% are older than 65. District nursing care in the Netherlands can be enlisted directly by referring physicians, paramedics, and patients. It is conducted by autonomous district nursing teams consisting of home care assistants (EQF-level two), nursing assistants (EQF-level three), vocational nurses (EQF-level four), and district nurses (EQF-level six) [27].

In the Netherlands, the Health Insurance Act (Zvw) mandates that all individuals aged 18 and older who work or reside in the country obtain basic health insurance. Although this compulsory insurance is generally affordable, the financial burden is distributed through various mechanisms, including healthcare allowances for low-income individuals. The basic insurance package covers reimbursement nursing and home care, without a deductible. Dutch district nursing care can also be provided under the Long-Term Care Act (Wlz) for adults needing 24/7 supervision or care availability, through an assessment of the Centre of Care Assessment (CIZ). In 2023, 15% of older adults with district nursing care received this care under the Long-Term Care Act. Therefore, for home dwelling older adults, this system ensures access to essential district nursing services based on a needs assessment [26]. Dutch district nurses are the gatekeepers of care at home under the Zvw and formally determine care [18]. This means they decide about district nursing care’s form, intensity, and duration during intakes and evaluations. In addition, they perform (complex) nursing care tasks. The care plan of the district nurses is located in their electronic patient record. Patients have access to this care plan. The Dutch Professional Association of Nurses and Caregivers (V&VN) states that district nurses are leaders in making decisions with older adults about physical, social, and emotional independent functioning [18]. SDM in healthcare is legally required in the Netherlands since January 2020 [27].

### Participant selection and recruitment

For district nurses, the inclusion criterion was: (1) employed as a district nurse at an organization affiliated with the Data Nurse project; (2) willing to find an older adult to participate in the study. The inclusion criteria for older adults were: (1) older than 60 years; (2) having two or more chronic conditions; (3) speaks Dutch or interacting with an interpreter; (4) receives care from the organizations within the Data Nurse project; (5) with or without an informal caregiver; (6) is receiving care for the first time or needs an evaluation of care. No exclusion criteria had been applied.

Participant recruitment occurred as follows: contact persons of the participating Data Nurse organizations received an informative email about the study. Contacts were asked to recruit district nurses. Participation was voluntary and requested through the organization’s online platform. Researcher SWH sent the recruited district nurses an email about the topic of the study and an information letter including a consent form for district nurses. These district nurses also received the inclusion criteria and an information letter including a consent form for participating patients. They were each asked to find one patient. The district nurses purposefully recruited patients who already received care [28] by explaining the study verbally and asking patients if they were available for video observations during an evaluation. New patients were asked if the intake of care could be videotaped. Upon confirmation, the information letter was given to patients. Before data collection, a consent form was signed by all participants. After digitalizing, the original (signed) consent forms were destroyed.

### Data collection

Recordings took place from December 2023 through September 2024. When participating district nurses identified a willing patient, they emailed the researcher (SWH) about the intake or evaluation date and time. It was also coordinated whether the recording would be on location or through the laptop’s camera (by using Microsoft Teams for recording). Therefore, although all intakes and evaluations took place at the homes of the older adults, the way of recording differed. This difference depended on the preference of the district nurses and older adults, and the feasibility of on-location recordings. For example, if an intake was announced shortly before start, the researcher (SWH) could not always record on location in time. After video recording, older adults, informal caregivers, and district nurses were given a short questionnaire (Additional file 1) to collect baseline data and information about the care request. Separately from each other, they completed this questionnaire. In addition, in consultation with older adults, the district nurses described the referrer’s care request from the electronic patient record.

### Development of the observational coding scheme for verbal communication in the district nurse-older adult/informal caregiver communication during SDM

To explore alignment of observed verbal communication during SDM in district nursing care with existing SDM models, two researchers (SH, MSc, registered district nurse with a PhD position, and RPL, PhD and expert on SDM) had defined an observational coding scheme for verbal communication during SDM. This scheme captures the verbal communication associated with key steps of SDM as described in established SDM models. Additional File 2. presents the components of this coding scheme. The scheme was based on “The Dynamic Model of Shared Decision-Making in frail older patients” [29] and “The Model for Goal-Based Shared Decision-Making” [2]), and the corresponding SDM preparation tool for older adults and their informal caregivers [3, 5].

### Development of the observational coding scheme for non-verbal communication

To explore the non-verbal communication during SDM, an observational coding scheme was developed for non-verbal communication (SWH). The scheme was based on an existing coding scheme to code non-verbal communication between nurses and older adults [30]. The following codes are included from the scheme: (1) making eye contact (interest); (2) nodding and shaking head affirmatively (kindness, interest, and encouragement to speak further); (3) smiling (forge a good relationship); (4) forward leaning (listening, involvement); (5) affective touch (affection and care); (6) instrumental touch (touch to perform a nursing task) [30]. The scheme was supplemented with one code: “co-speech gestures”. Spontaneous co-speech gestures are hand movements that facilitate speech organization and/or messages presented through speech [31, 32]. This code was added (SWH, RPL) since reviewing the recordings revealed that district nurses often supported verbatim explanations with gestures. Additional File 3. shows this coding scheme for non-verbal behavior in district nurse-older adult/informal caregiver communication and a description of the observations.

### Data analysis

Using audio/video observations during intakes and evaluations of care, performed by district nurses, we analyzed the district nurse’s interaction with the older adults regarding moments of decision making during intakes or evaluations at older adults’ homes [33]. Verbal and non-verbal communication, related to SDM on interventions to support independent functioning, was analyzed. Verbal communication was qualitatively analyzed. Non-verbal communication was quantitatively analyzed.

### Analysis of verbal communication

Verbal communication was analyzed thematically with MAXQDA-2022 [34]. This data analysis followed an inductive-deductive (hybrid) approach. This means that the analysis began with an inductive process, where themes were derived directly from the raw data without preconceived categories, allowing the data to speak for itself. Subsequently, to explore how shared decision-making by district nurses aligns with existing person-centered SDM models, a deductive approach was applied. In this approach, the observational coding scheme for verbal behavior in district nurse–older adult/informal caregiver communication (Additional File 2) was employed as a conceptual framework to organize the inductively derived themes. This approach facilitated an examination of how these themes align with and contribute to existing person-centered SDM models [35].

Thematic analysis was conducted using the following steps by Braun and Clarke [36]: First, familiarity with the collected data was reached by reading and re-reading the transcripts (SWH, DO). Second, transcripts were coded through open coding (SWH, DO), compared, and discussed until consensus (SWH, RPL). Third, themes were formulated in a codebook (SWH, RPL) and framed in the observation scheme for verbal communication. Fourth, themes were discussed and adjusted (SWH, RPL). Fifth, specifics of each theme were refined, clear definitions were generated, and names were made for each theme. Data saturation was reached if transcripts revealed no new themes. Saturation was discussed with SWH and RPL. Sixth, a final code tree was created with inductively found themes linked to deductive codes (Additional File 4). Participant quotations were presented to illustrate the themes and findings. A participant number identified each quotation.

### Analysis of non-verbal communication

Non-verbal communication was analyzed with Behavioral Observation Research Interactive Software (BORIS), version 8.21.10 [37]. It was coded using the coding scheme for non-verbal communication (Additional File 3) and analyzed quantitatively.

### Reflections of participants on the summarized results

After the analysis, SWH emailed a summary of the results from the verbal and non-verbal communication to the participating district nurses. The participants’ reflections on the summarized results (written comments) were emailed to SWH. SWH summarized these reflections and discussed them with RPL, BB, and NB. The summary of the participants’ reflections are presented in the result section.

### Rigor

Data trustworthiness criteria were used in this study [38]. To ensure credibility, the codes had been extensively pre-discussed with an expert in the field of SDM (RPL). To ensure transferability, the study setting was accurately described and study participants reflected patients from district nursing practice. Extensive logs of raw and transcribed data were kept to ensure reliability. To ensure confirmability, the summarized results were checked. Participants reflected on the summarized results. Open codes were created with two researchers (SWH, DO), and themes were also constructed with two researchers (SWH, RPL). To avoid themes being driven by the researcher’s experience or perception, themes were validated by comparing them with the transcripts (SWH, RPL). For reliability of non-verbal observations, an intra-rater agreement was measured.

### Ethics

Before the commencement of the study, approval (research protocol number 2024.0653) was waived by the Medical Research Involving Human Subjects Act (WMO) of the Amsterdam University Medical Center (AUMC) (Additional file 5). The Declaration of Helsinki has been used to follow ethical guidelines [39]. Informed consent was obtained of all participants in the study. Participants were assured that all personal data had been coded, rendering them untraceable. Video recordings and transcripts were securely stored on a protected server at the Amsterdam University Medical Center, with access strictly limited to the research team.

## Results

For each recording, the characteristics of the participants and observations were described (Table 1 and 2). A total of 26 eligible district nurses were recruited. Ten nurses dropped out: six provided no reason, and four announced they could not find a patient. In total, 16 were filmed with an older adult during an intake or evaluation. Table 1. shows that the average age of older adults was 79.4 years (range 65–90) and the average age of district nurses was 37.8 years (range 24–63). Recordings involved seven intakes and nine evaluations at the homes of older adults. Five recordings were live, and eleven were online via Microsoft Teams. Table 2. shows that the intakes took on average 33.64 min and evaluations 32.60 min. Older adults used most words on average. Informal caregivers used the fewest words on average. The brief questionnaire for participants (Additional File 1), that participants received after filming, revealed that patients often described a different care need than their informal caregiver, or district nurse.

**Table 1:** Characteristics of participants and description of the care need according to referrer, patient, informal caregiver and district nurse

Obs	Age & sex pt	Age & sexDN	Marital status pt	IC, relation	Tasks IC	Health and welfare problems pt	Days of DC per week	Referrer and CN according to referrer	CN accor-ding to pt	CN accor-ding to IC	CN according to DN	Years of experi-ence DN
1.	80 m	25 f	married, with partner	no		renal failure, cerebral infarction, diabetes	1	hospital: wound care, bladder catheter care	bladder catheter care instruction		support independence, lifestyle coaching	4
2.	75 m	30 f	married, with partner	yes: husband	house keeping	vertebral and rib fracture	7	nursing home: ADL	ADL after fall from stairs	ADL after fall from stairs	GR, support independence, wounds, weight control	10
3.	78 m	57 f	married, with partner	yes: wife	ADL, house-keeping, transport	heart failure, pressure ulcers, renal failure, prostate and pancreas cancer, diabetes	1	GP: wound care	wound care	assis-tance IC tasks	assessment over-load IC, wound care oncological disease coun- seling	38
4.	87 f	25 m	widow alone	yes: daughter	administrator, daily phone calls, weekly visits and grocery shopping	dementia, social isolation, skin rashes	7	GP: ADL	Dementia assistance	ADL, IADL.	ADL, nutritional and medication control	4
5.	80 m	28 f	widow alone	yes: daughter	grocery shopping	arthrosis, diabetes	7	hospital: GR, heparin injection, ADL	GR	GR	GR, wound care, stimulating independence	10
6.	75 f	59 f	widow alone	yes): son	grocery shopping	rheumatoid arthritis, so-cial isolation	7	general practitioner: ADL	ADL		support of independent living	30
7.	79 m	36 f	widow alone	yes: son	grocery shop- ping, transport	back pain with reduced mobility	7	nursing home: ADL	ADL, applying Fentanyl patch		ADL applying Fentanyl patch	12
8.	86 f	45 f	widow alone	yes: son	grocery shopping and trans- port	cardiac failure	7	hospital: ADL, wound care	wound care		medication control, wound care, support independence	27
9.	80 f	24 f	widow alone	no		Parkinson disease, social isolation	4	General practitioner: ADL	ADL		ADL, help finding social activities	3
10.	83 f	38 f	un-married, with partner	yes: friend	house keeping	arthrosis, back stenosis	3	self-requested DN care	ADL		support of independence	15
11.	75 f	30 f	un-married, with partner	yes): friend	gardening, cooking	Lung cancer, COPD	3	self-requested DN care	lung drain care		lung drain care	10
12.	65 m	63 f	married, with partner	yes): wife	overall guidance	dementia social isolation	1	GP: coordination of dementia	un-known	CM demen- tia	CM support dementia	13
13.	90 f	56 f	widow alone	no		wounds, bruised ribs	7	nursing home: ADL and wound care	ADL and wound care		ADL, wound care	10
14.	69 f	36 f	widow alone	yes: son	transport, gardening	heart failure	7	nursing home: ADL after femoropopliteal bypass.	ADL		ADL, wound care, treating fungal infection.	12
15.	81 m	25 f	widow alone	no		throat cancer, mourning	7	hospital: tube feeding	tube feeding		bereavement support, tube feeding, giving medication	1
16.	87 f	28 f	Un-married, alone	no		low vision, heart failure	7	GP: oedema bandaging	training on compression stocking tool		support of independence.	6

**Table 2:** Characteristics of the observations

Recording	Conver- sation in minutes	Informal caregiver attended	Total number of words district nurse (%)	Total number of words older adult (%)	Total number of words informal caregiver (%)	Number of times spoken district nurse	Number of times spoken older adult	Number of times spoken informal caregiver
1. Evaluation live	33.37	-	581 (19.5%)	2405 (80.5%)		92	91	
2. Intake live	30.18	yes	2086 (47.1%)	1800 (41.0%)	319 (11.9%)	155	121	82
3. Intake live.	27.35	yes	1553 (38.2%)	2005 (49.4%)	501 (12.4%)	122	102	57
4. Intake live	33.30	yes	1854 (43.9%)	1552 (36.8%)	814 (19.3%)	107	118	28
5. Evaluation live	31.23	yes	2561 (61.5%)	802 (19.2%)	804 (19.3%)	120	96	64
6. Intake online	10.00	no	839 (71.1%)	288 (28.9%)		63	58	
7. Evaluation online	41.02	no	989 (30.0%)	2312 (70.0%)		134	134	
8. Intake online	48.25	no	2735 (46.8%)	3113 (53.2%)		159	159	
9. Intake online	36.06	-	1741 (72.1%)	673 (28.9%)		111	111	
10.Evaluationonline	32.46	no	1512 (50.3%)	1496 (49.7%)		106	105	
11.Evaluation online	52.06	no	1874 (24.4%)	5807 (75.6%)		214	215	
12.Evaluation online	50.00	yes	1524 (27.1%)	2703 (48.0%)	1401 (24.9%)	63	45	33
13.Evaluation online	24.53	-	1970 (53.3%)	1723 (46.7%)		159	159	
14.Intake online	50.34	no	2781 (38.2%)	4497 (61.8%)		310	298	
15.Evaluation online	19.42	-	1065 (41.5%)	1395 (58.5%)		62	61	
16.Evaluation live	9.33	-	589 (44.5%)	734 (55.5%)		60	64	
Total	528.09	5	26,254 (41.4%)	33,305 (52.5%)	3839 (6.1%)	2063 (48.8%)	1937 (45.8%)	231 (5.4%)

Inductive analysis of the verbal communication revealed eight themes (Table 3.): being informed at start of the conversation; initiating the conversation; exploring care needs; demonstrating attentiveness to the older adult; identifying care problems and care goals; presenting and discussing options; agreeing on district nursing care, and concluding the conversation. Code saturation was reached after the twelfth recording. However, 16 interviews were needed to reach meaning saturation, where we developed a richly textured understanding of the themes [40]. Deductive analysis revealed the alignment of the inductive themes with the deductive codes representing verbal communication, as shown in existing SDM models. Dialogue quotes will describe the older adult with “OA”, the informal caregiver with “IC”, and the district nurse with “DN”.

The analysis of non-verbal communication supported the findings from the verbal communication, indicating that district nurses demonstrated attentiveness and actively encouraged older adults to express themselves. The textual comments on the findings revealed that district nurses emphasized the significance of engaging older adults in decision-making and valued the establishment of a trusting professional relationship. However, they expressed skepticism regarding the extent to which such trust could be fully realized within the framework of a SDM model.

**Table 3:** Deductive codes, inductive themes and sub-themes of the study

Deductive codes	Inductive themes	Inductive sub-themes
Preparation prior to the conversation	1.Being informed at start of the conversation	
Team talk	2. Initiating the conversation	1. Explaining the intent of the conversation
		2. Structuring the conversation
		1. Introducing information sources and discussion topics
	3. Exploring care needs	
	4. Demonstrating attentiveness to the older adult	4. Exploring what matters to older adults
		5. Having conversations at the patient’s level
Goal talk	5. Identifying care problems and care goals	
Option talk	6. Presenting and discussing options	6. Discussing goal-oriented care options
		7. Involving informal caregivers in SDM
Decision talk	7. Agreeing on district nursing care	8. Making shared decisions on independent management of medical conditions
		9. Making shared decisions on district nursing care related independence
		10. Making shared decisions on independent functioning
		11. Recognizing older adults as the primary decision-makers
Evaluation talk	8. Concluding the conversation	12. Evaluating decisions made

### Verbal communication

#### Deductive code: Preparation prior to the conversation

- Patient formulates personal goals, and related health and care goals.
- Healthcare professional identifies history of care and care problems.

#### Theme 1. Being informed at start of the conversation

Many older adults had not considered their future care needs for independence, and district nurses typically did not explore their personal goals or preferences. Instead, district nurses focused on immediate care needs based on information from referring professionals. The following exchange illustrates this approach:

DN: “Your GP informed me about the condition of your husband, and told me that you need help with informal care tasks. Can you tell me more about this care request?”

IC: “I think I need more help with household chores?”

DN: “Do you also need support caring for your husband, so you can stay independent?”

OA: “I have pressure ulcers; it is overwhelming for her.”

IC: “I am not sure what else I need.” (5)

#### Deductive code: Team talk

- Healthcare professional introduces the decision-making process
- Healthcare professional identifies the discussion partner

#### Theme 2. Initiating the conversation

##### Sub-theme 1. Explaining the intent of the conversation

District nurses usually started the intake or evaluation by explaining the reason of the conversation or asking if the older adult was aware of it. At the start of intakes they often indicated that the conversation was initiated following a physician referral. At the beginning of evaluations, they often mentioned that the evaluation was routinely scheduled. While nurses often emphasized the involvement of the older adult in care planning, they rarely clarified how preferences should be communicated or how decisions would be made.

DN: “You know the purpose of why I am here?

OA: “Yes.”

DN: “This intake is about the care we provide. I will also ask what care you expect from us. I will ask questions and make practical suggestions. We can talk about that.

OA: “Yes.”

DN: “Following the request from the hospital after the surgery, I have to ask what care you expect from us.” (8)

The following dialogue illustrates how district nurses acknowledged older adults’ preferences and reinforced their decision-making role in selecting appropriate interventions:

DN: “We will evaluate your device. It should suit you. I asked the occupational therapist yesterday to find an appropriate assistive device.”

OA: “Thank you, I like that. The assistive device I have now does not work for me. I want one I can get away with.”

DN: “It must be consistent with your wishes.” (16)

##### Sub-theme 2. Structuring the conversation

Conversations varied in structure and were primarily shaped by the input of older adults and the district nurse’s responses to their input. The district nurses did not structure the conversation with delineated stages, such as a team talk, goal talk or a decision talk. In two evaluations, district nurses developed a questionnaire informed by documentation from the electronic patient record.

DN: “We evaluate once a year in our regular way.”

OA: “I know that by now.”

DN: “I prepared some questions by the care plan and reports from the past. That is my structure for the interview. I always ask first: ‘How are you doing?’” (11)

##### Sub-theme 3. Introducing information sources and discussion topics

District nurses often began conversations by introducing directional sources of information for intake or evaluation. Information from care plans was central during evaluations, while intakes relied on information from medical records, transfer documents, and medication reviews. In most intakes, care requests from general practitioners, hospitals, or nursing homes were identified as the main guiding inputs.

DN: “We are addressing the hospital’s care request, which includes daily help with compression stockings, undergarments, and wound care.”

OA: “Okay.” (7)

DN: “Did the hospital tell you what we should do and what you can or cannot do?” (5)

Moreover, district nurses introduced various aspects of care, including the financing of district nursing services, ergonomic working conditions in patients’ homes, the team’s accessibility, allocated care hours, and the involvement of medical or nursing students. However, generally, they did not address older adults’ central concern regarding which topics were open for discussion within the scope of district nursing care. The following exchange highlights how district nurses often focused on practical and organizational aspects of care, while older adults sought clarity on the scope of support available:

OA: “I have never experienced this situation before. I do not know what to expect. What can district nurses do?”

DN: “Well, we are financed from your basic insurance. You will not get a bill from us. We will make a care plan as a result of this conversation.” (14)

#### Theme 3. Exploring care needs

Overall, district nurses did not provide a clear explanation of the scope or nature of district nursing care during intakes or evaluations. However, many older adults and informal caregivers struggled to articulate their specific care needs. When nurses posed open-ended questions, such as asking what care was needed, older adults frequently appeared uncertain or were unable to respond. Several indicated they had not yet reflected on the type of care they might require and expressed a lack of understanding regarding the services district nursing could offer. Rather than clarifying the scope of care, district nurses often repeated the question or encouraged older adults to reflect further on their needs, without offering additional guidance. For example, one informal caregiver who emotionally expressed (by crying) that she was unable to continue caring for her husband was unable to articulate her care needs for district nursing care. The district nurse nevertheless encouraged her to articulate her care needs.

DN: “How can we support you? Do you have an idea of that?”

IC: “That is difficult.”

DN: “Yes, I also understand that is difficult. But I can see that you are troubled. Think about how we can support you anyway.”

IC: “I have never actually done that.”

DN: “No, that is why I think it overwhelms you. But I can see that it is high on your mind. Please think about it, dear. It has been going on for a long time, I think?” (3)

District nurses tended to respond promptly to care needs once these were expressed.

OA: “If I am independent, will I never see a district nurse again? I would appreciate occasional contact, even just a phone call. My vision is poor. Can I consult you sometimes?”

DN: “I understand, I will discuss with the team how we can manage that.” (16)

#### Theme 4. Demonstrating attentiveness to the patient

##### Sub-theme 4. Exploring what matters to older adults

Overall, district nurses demonstrated a strong commitment to older adults and informal caregivers. For instance, all district nurses expressed empathy and compassion for the patients’ circumstances.

DN: “It has been a very emotional time for you, hmmm.”

OA: “Yes…”

DN: “Hmmm, wife and daughter-in-law passed away in nine weeks, very intense.” (7)

The majority of district nurses inquired about the recent well-being of the older adult. Older adults who had experienced a significant event, such as a hospitalization or the death of a partner, responded in depth. District nurses generally listened attentively, responded thoughtfully to the patients’ accounts, and encouraged further discussion.

DN: “Do you have something to live for every day?”

OA: “Yes, most of the time. I am rarely alone; if I am, I often do not mind.”

DN: “Hmm, does it give peace of mind?”

OA: “It gives me time to do my creative things. Reading a book, this kind of thing.”

DN: “Hmmm, anything else you want to say about this?” (11)

##### Sub-theme 5. Having conversations at the patient’s level

Overall, district nurses communicated at the patient’s level and routinely verified understanding. However, some district nurses employed jargon that appeared unclear to the patient.

DN: “I am going to finish this interview. I am going to sign you into the system and take care of everything behind the scenes.” (5)

Two evaluations involved older adults with dementia and their informal caregivers. The district nurses adapted their communication to the cognitive level of the older adults. They clearly articulated and ensured understanding through checks for comprehension.

DN: “Do you wish to meet other people? To be among people and create things? Do you understand? ”

OA: “For me, it is not necessary to craft in my neighborhood.”

IC: “It is also for me. So I can be free for a while.”

OA: “Yes, for her it is good.” (12)

#### Deductive code: Goal talk

- Healthcare professional identifies patient values and fundamental goals
- Healthcare professional identifies the capacities of the patient
- Healthcare professional identifies care problems, and related care goals
- Healthcare professional identifies the prioritization of care goals

#### Theme 5. Identifying care problems and care goals

Overall, care goals were not explicitly elicited or defined with older adults during intakes or evaluations. Most older adults shared detailed information about challenges to their independence across multiple domains, and district nurses generally allocated time to discuss these concerns. However, systematic identification and prioritization of independence needs, independence capacities, as well as the formulation of specific care goals, were often lacking. Additionally, many district nurses employed a solution-focused approach, promptly suggesting interventions and asking for solutions in response to the many independence challenges reported by older adults. Furthermore, in many conversations, the focus of the interaction remained primarily on providing a supportive, listening presence.

OA: “There are many inconveniences I must learn to live with.”

DN: “Yes, yes.”

OA: “I deal with it my way, but I have hardly walked since I left the hospital. My walker was converted for my wife in my absence. She had back pain.”

DN: “Yes, yes, yes.”

OA: “She needed it for groceries.”

DN: “Hmmm.” (1)

Only in brief intakes or evaluations where few care problems were identified did some district nurses explicitly inquire about the care goal.

DN: “If I understand correctly, your overall care goal is to get your compression stockings on and off yourself.” (16)

Sometimes, after an evaluation was completed, a Patient Reported Experience Measure (PREM) questionnaire was administered. This included questions assessing whether the provided care aligned with the older adult’s personal life goals and goals related to independence.

DN: “Does the care fit the way you want to live? When the care worker visits you, are your wishes and activities taken into account?” (4)

DN: “Does care enable you to do activities that are important to you, despite your illness or condition?” (6)

#### Deductive code: Option talk

- Healthcare professional summarizes and offers choices to address the care goals
- Healthcare professional discusses personalized care options and patient participation

#### Theme 6. Presenting and discussing options

##### Sub-theme 6. Discussing goal-oriented care options

As care goals were infrequently established, many district nurses proposed optional interventions to support independence without prior agreement on the older adults’ goals. Although older adults often emphasized that interventions should align with their preferences, these preferences were not consistently elicited.

DN: “You have not used the exercise bike since discharge. Would you like to try cycling at the physio?”

OA: “I do not think it would help.”

DN: “It works the same muscles as the bike in the nursing home. We could also rent a pedal bike for your chair.”

OA: “No, it has to be a real benefit for me.” (7)

In addition, some district nurses determined interventions without consultation. This was particularly the case in cases of suspected incorrect medication use.

DN: “These are your medicines?”

OA: “No, these are other medicines.”

DN: “I see you have your own schedule. I will add you to our online medication app, in which we sign off medications we give to patients.” (5)

In addition, some district nurses mentioned that the goal of care was to implement the agreed-upon interventions.

DN: “The goal is to provide personal care and email the occupational therapist to assess home modifications with you.” (2)

##### Sub-theme 7. Involving informal caregivers in SDM

Moreover, when informal caregivers were present during the interview, district nurses often involved them by discussing their potential role in supporting the older adult’s independent functioning, for example, by coordinating with other healthcare professionals.

“DN: Can you call in the physio for exercise and the GP for the medication sachet system? ”

“IC: Yes.” (2)

Older adults varied in their perspectives on involving informal support networks. District nurses generally did not press for the involvement of such networks when it became apparent that this option was not open or feasible for the older adult.

DN: “How can your network help in housekeeping?”

OA: “Of course, the well-known story of networks, friends, and neighbors. Well, my daughters are busy with work and kids. I cannot and do not want to ask them to do my housekeeping on weekends.”

DN: Have you contacted the municipality about domestic help? Is the number of hours expandable?” (11)

#### Deductive code: Decision talk

- Healthcare professional connects to patient’s values and goals in care
- Healthcare professional and patient make shared decisions on nursing interventions

#### Theme 7. Agreeing on district nursing care

Although district nurses did not introduce topics on which decisions could be made, decisions were made on many topics and the decisions were often focused on independent functioning. The three main topics are outlined in the sub-themes below.

##### Sub-theme 8. Making shared decisions on independent management of medical conditions

Decisions on this topic were mainly made regarding independent medication use, wound management, and instructions from referrers. For instance, most older adults agreed to follow prescribed medication schedules, and in cases of noncompliance, district nurses emphasized the importance of adherence.

“DN: You really should keep taking the pain medication anyway. I suggest you follow these prescriptions.” (5)

##### Sub-theme 9. Making shared decisions on district nursing care related independence

Care decisions often aligned with the referrer’s requests, particularly regarding “district nursing care-related independence,” such as participation in urinary catheter care or using assistive devices. In addition, care visit times were adjusted to older adults’ preferences. To optimize care-related independence, district nurses usually recommended assistive devices, as donning and doffing aids for compression stockings, eye drop glasses, shower chairs, bed rails, toilet chairs, wall brackets, medication dispensers, key lockers, and walkers were often discussed. Older adults preferred district nursing care if they were unsure about using an assistive device or lacked information on its use.

DN: “About the eyedrops…The care plan suggests considering eye drops, so you can drip yourself. Did you try it?”

OA: “No.”

DN: “No? Would you like to learn it?”

OA: “I do not think I can.”

DN: “We will practice first. Put on the glasses, place the bottle in the holes, and just squeeze—it will drop in.”

OA: “Well, it does not matter how I do it. I cannot drop.” (10)

Using assistive devices appeared difficult for many older adults. The teaching of handling assistive devices was often discussed.

DN: “How are you experiencing the care so far?”

OA: “Fantastic, but this device for my compression stockings is so stiff—many older adults must struggle with it. We have self-driving cars; surely there is a better solution?”

DN: “Yes. What do you run into the most with this device?”

OA: “I cannot get the stocking over the heel.”

DN: “You also have poor vision, that also plays into it?”

OA: “Yes.”

DN: “The occupational therapist is looking for solutions with you?”

OA: “Yes.” (16)

The advantages and disadvantages of assistive devices were frequently discussed. Older adults most commonly identified ‘maintaining independence from care’ as the principal benefit of using such devices. To illustrate this, one district nurse emphasized this benefit when discussing an assistive device with an older adult:

DN: “If you drip your eyes yourself, we do not have to visit in the afternoon and evening.”

OA: “Well…”

DN: “Then you have more freedom, and we do not ring your doorbell in the evening.” (10)

Furthermore, the assessed degree of independence, from hospital or nursing home transfer forms, was often critically discussed. Moreover, district nurses often emphasized that safety was paramount when functioning independently.

DN: “The nursing transfer form states that you take your medication yourself?”

OA: “Yes, I can do that myself. Above all, I like it a lot better.”

DN: “That you are capable of doing it, is also important.” (7)

##### Sub-theme 10. Making shared decisions on independent functioning

Addressing problems with independent functioning was a central topic in all intakes and evaluations. Many decisions were made on this topic. Generally, district nurses did not systematically assess independent functioning by a questionnaire, but discussed independent functioning at the moment the older adult highlighted problems with independent functioning. Many older adults reported condition-related independence problems in physical, social, and environmental areas. Most district nurses holistically offered solutions to these problems, based on their professional expertise and the patient’s problems. Decisions included interventions such as nursing home admission, day care, home modifications, and support from domestic or informal care. For example, one district nurse discussed potential support with an older adult:

DN: “How do you feel about seeing an occupational therapist?”

OA: “That is fine.”

DN: “You mentioned not wanting more care. This could help you stay independent.”

OA: “I dress myself, using a chair in the shower.”

DN: “A shower chair helps prevent falls—you are clearly quite unsteady.” (9)

Moreover, district nurses frequently referenced their observations of the level of independent functioning during care interactions. They did not hesitate to provide urgent recommendations when safety concerns were identified.

OA: “I use the stool to dry my feet.”

DN: “That wobbly stool? That is not safe.”

OA: “Really?”

DN: “Yes, it is unstable. Do you still have the shower chair?”

OA: “No, they took it away.”

DN: “Maybe your daughter can help get one. There are small, safe stools. Yours is not firm, and if you get light-headed, it could be dangerous.” (13)

##### Sub-theme 11. Recognizing older adults as the primary decision-makers

Decisions were generally made without reference to predetermined goals. When informal caregivers were present, their perspectives on the proposed interventions were considered; however, the final decision rested with the older adult.

“DN: I can sign you up for the night team.”

“IC: You might do that.”

“OA: No, not necessary.”

“DN: Okay.” (5)

Although the older adults made the final decisions, some were influenced by the district nurses. As shown in the quotes below, the district nurses’ directive approach was often grounded in their prior professional experience.

DN: “Of course you can wash and dress yourself. However, this week is the first week at home, and from my experience, we therefore should keep an eye on you.” (2)

DN: “Many say they have no pain on the first day home, but the first two weeks are crucial, especially for those living alone. If things go well by next Friday, we will reduce the care.” (5)

#### Deductive code. Evaluation talk

- Healthcare professional initiates an evaluation of the decision and SDM process

#### Sub-theme 12. Evaluating decisions made

At the end of the conversations, most district nurses did not clarify what care the older adults and informal caregivers expected to receive from district nursing care. For example, older adults were not asked to summarize the decisions in their own words.

“DN: If you have no further questions, we will stop the conversation.” (7)

Some district nurses summarized the decided care in their own words and asked if the older adults and informal caregivers had further questions. If additional care needs were identified or if the intervention was unclear, this often led to new decisions, as the quotes below illustrate. However, the decision-making process itself was never formally evaluated.

DN:” I will conclude this conversation unless you have more questions?”

OA: “Can you also measure my calves? I need new compression stockings.”

DN: “Oh. Do you have a tape measure? I think you will need help putting on the new stockings, as they are quite firm.” (9)

DN: “We have agreed on assistance with showering twice a week, and washing on the bed on other days. Do you agree?”

IC: “She washed independently at the sink in the nursing home.”

DN: “Oh, not on the bed? We have to help with washing in the kitchen?”

OA: “Yes.”

DN: “Do you have good footwear?”

OA: “Yes, slippers with good soles.”

DN: “We have our fall prevention team. Should I ask them to visit?”

OA: “Okay.” (2)

**Table 4:** Quantitative description of analyzed non-verbal communication of district nurses

Recor-ding	Making eye contact (% of time)	Nodding and shaking head affirmatively (% of time)	Smiling (% of time)	Forward leaning (% of time)	Affective touch (% of time)	Instrumen-tal touch (% of time)	Explaining with gestures (% of time)
1	2 (99.9)	156 (28.5)	11 (0.7)	3 (0.2)	0 (0.0)	0 (0.0)	11 (0.7)
2	10 (98.5)	107 (7.0)	20 (1.3)	6 (0.4)	4 (0.3)	1 (0.1)	52 (3.3)
3	14 (98.7)	119 (8.3)	23 (1.6)	3 (0.2)	1 (0.1)	1 (0.1)	51 (3.6)
4	63 (82.9)	127 (7.3)	38 (2.4)	0 (0.0)	0 (0.0)	0 (0.0)	9 (1.0)
5	38 (70.8)	57 (6.7)	7 (0.6)	7 (0.4)	0 (0.0)	0 (0.0)	37 (3.3)
6	8 (96.9)	32 (16.1)	17 (6.1)	14 (3.0)	0 (0.0)	0 (0.0)	13 (7.1)
7	11 (97.2)	120 (11.4)	26 (1.8)	8 (0.4)	0 (0.0)	0 (0.0)	13 (1.4)
8	25 (95.8)	120 (12.2)	12 (1.0)	2 (0.2)	0 (0.0)	1 (0.1)	21 (2.0)
9	3 (99.6)	58 (4.4)	8 (0.7)	3 (0.2)	0 (0.0)	0 (0.0)	11 (0.9)
10	1 (100)	33 (4.5)	13 (57.0)	34 (41.7)	1 (0.1)	0 (0.0)	36 (57.7)
11	67 (88.3)	192 (11.1)	6 (0.3)	0 (0.0)	0 (0.0)	0 (0.0)	9 (0.4)
12	9 (99.5)	57 (2,5)	41 (10.7)	13 (2.9)	5 (0.2)	0 (0.0)	24 (1.7)
13	13 (98.6)	58 (35.4)	12 (51.9)	17 (25.6)	0 (0.0)	0 (0.0)	9 (0.8)
14	8 (95.1)	92 (13.5)	18 (0.7)	4 (12.3)	0 (0.0)	5 (0.4)	14 (0.7)
15	2 (98.7)	137 (13.4)	16 (1.4)	2 (45.0)	2 (0.3)	0 (0.0)	6 (0.5)
16	1 (100)	39 (8.8)	4 (0.5)	1 (96.8)	0 (0.0)	0 (0.0)	1 (0.1)
Total	275 (94.4)	1471 (11.3)	272 (7.9)	117 (8.9)	13 (0.1)	8 (0.1)	317 (5.1)

### Non-verbal communication

Analysis of non-verbal communication (Table 4.) revealed that district nurses expressed non-verbally that they were friendly, interested in older adults, and encouraged them to continue talking. This was particularly evident in making eye contact and nodding and shaking affirmatively.

On average, eye contact was made for 94.4% of the conversation duration and ranged from 70.8 to 100%. The observations showed that the eyes of district nurses particularly deviated from those of older adults when looking at the electronic patient record on the laptop. The percentage of time nodding and shaking affirmatively from the total length of the conversation ranged from 2.5 to 35.4%. Since the nods mainly were brief moments, affirmative nods averaged 11.3% of the time per conversation. The observations showed that nodding took over talking during emotional conversations, for example, about death and loss. Three of the sixteen observations (observation 3,14, and 16) were re-coded by researcher SWH for intra-rater reliability. They respectively had a Cohen’s Kappa of 0.95, 0.95, and 0.97, representing excellent agreement.

### Textual reflections on the summarized results

Fourteen of the sixteen participating district nurses provided feedback via email regarding the summarized results of the observations, offering additional textual comments. In general, participants acknowledged current decision-making practices within district nursing care as reflected in the summarized results. Additionally, they expressed diverse perspectives regarding the implementation of a structured SDM model (points 1 and 2). In summary, the following key responses were identified:

1. A structured Shared Decision-Making (SDM) model, with a focus on goal setting, is essential for district nursing care and should be developed.
2. Let older adults determine the structure of the conversation. A predetermined model is presumably a quick checklist with no room for personal input and may limit the discussion of personal views on health problems. Moreover, it could prevent the building of a trusting relationship.
3. Building trust is key to decision-making, especially with older adults who have low health literacy, reduced decision-making ability, or dementia. To build trust, allowing time to explain “what’s going on” is essential.
4. Assessment questionnaires to evaluate independent functioning should be used more frequently.
5. District nurses should provide clearer explanations of decision-making topics.
6. Older adults should be asked to prioritize their care needs since they have many needs. It should be added to the decision-making process.
7. Due to the length of intakes and evaluations, it is often too challenging for most older adults to recap the decisions made.

## Discussion

We conducted a video observation study. To the best of our knowledge, this is the first study to observe how district nurses make verbal and non-verbal person-centered shared decisions about supportive interventions for independent functioning with home-dwelling older adults. Eight themes emerged from the analysis of the verbal communication: being informed at start of the conversation; initiating the conversation; exploring care needs; demonstrating attentiveness to the older adult; identifying care problems and care goals; presenting and discussing options; agreeing on district nursing care, and concluding the conversation. The coding scheme of the quantitative analysis of the non-verbal communication comprised: making eye contact; nodding and shaking head affirmatively; smiling; forward leaning; affective touch; instrumental touch, and explaining with gestures.

Overall, district nurses seemed to make many decisions with older adults about interventions that support independent functioning. However, elements from SDM models were often not present. For example, no preparatory step was performed with the patient prior to intake or evaluation. In addition, elements beyond the existing SDM models seemed necessary. For example, “introduction of the healthcare professional” and “introduction of the topics of decision” seemed to be necessary elements in the decision-making process. The district nurses we filmed did not explain to older adults what decisions could be made and how those decisions could be made. Subsequently, many older adults had difficulty indicating (independence-related) care needs. Still, older adults raised a lot of independence issues. Although most district nurses listened attentively, they did not filter care goals from the older adults’ information, and tended to offer solutions immediately. As a result, options were offered that were not based on care goals. Moreover, independence was usually approached as care-related independence, for example, the degree of ability to dress independently if the referrer’s care request was assistance with dressing. Our results lead to the following two key discussion points:

First, the observation that district nurses did not use SDM models adds to earlier studies showing that SDM models are poorly covered in the education and practice of district nurses [16, 41, 42]. In our study, district nurses allowed older adults to determine the course of the conversation and gave them ample time to elaborate on problems they were facing. For example, verbal communication, non-verbal communication, and reflections on the summarized results indicated interest of district nurses in older adults’ health and welfare problems and encouragement to talk about these problems. Previous research showed that district nurses value investing time in building a relationship of trust and respect [20]; therefore, they allow the patient to speak out [41, 43]. Because the older adults determined the conversation, the conversations seemed unstructured. In the textual reflections on the summarized results, several district nurses suspected that a structured approach to shared decision-making would interfere with building a trusting relationship. This is inconsistent with the literature indicating that a trusting relationship is an important premise for shared decision-making in SDM models [20]. However, the older adults observed in this study were living with multiple health conditions. This population, in particular, requires a stepwise approach to decision-making, emphasizing goal setting, to ensure that care goals are not overlooked [44]. In addition, previous studies have demonstrated that older adults with MCC benefit from a clear explanation of the SDM process and an explicit invitation to participate in decision-making [45].

Second, independence needs and capacities were not systematically assessed, and independence goals were not explicitly discussed or prioritized. Instead, independence problems were discussed when older adults mentioned them, but many district nurses did not translate them into care goals. Furthermore, the themes revealed that older adults did not know what to expect from district nursing care and had difficulty describing their independence needs. In addition, care request-related independence was mainly discussed. However, previous research emphasizes the importance of exploring independence goals, as older adults rarely express them and medical referrals may overlook independence issues [44, 46, 47]. Especially for older adults with multiple conditions, it is vital to discuss their prioritized multiple (independence) problems, goals, and related care needs [14, 47]. Recent research showed that a lack of person-centered SDM was caused by dissonance in understanding the concept of independence between older adults and district nurses, because independence goals were not shared [41]. They found that older adults primarily defined independence as “the ability to self-determine and make decisions,” whereas district nurses associated independence more with “not being housebound.” Our results support these earlier findings. For example, older adults reported fewer independence-related care needs than district nurses, yet this discrepancy was not addressed or discussed during the conversations. The failure to establish care goals with patients contradicts the nursing process, in which care goals serve as the foundation for nursing interventions [48]. In addition, district nurses frequently employed a solution-oriented approach, often offering assistive devices when older adults raised concerns about independence. These decisions were made without a formal assessment of the individual’s independence-related needs. However, the selection of assistive tools could be more effectively informed through a systematic and structured assessment process [49]. Furthermore, the lack of assessment of care goals contrasts with principles of effective communication. Effective communication by district nurses enhances person-centered care and is especially crucial amid current healthcare workforce shortages. Minimizing unnecessary time during intakes and evaluations supports this efficiency. While observations indicated that many nurses primarily listened without guiding discussions, maintaining focus on older adults’ care goals is essential for improving both the efficiency and quality of consultations [46].

### Implications

The following implications emerged from the themes:

First, a person-centered SDM model should be developed specifically for district nursing care, in co-creation with district nurses and older adults with MCC to ensure alignment with decisions supporting independence. Building a trusting relationship should be a distinct, co-developed step. Additionally, given older adults’ limited awareness of decision-making possibilities, an introductory step is essential.

Second, given district nurses’ unfamiliarity and ambivalence toward conversational structures, a cultural shift is needed to implement SDM. Strategies should raise awareness that SDM models help build structured, trust-based relationships essential for person-centered care [2, 50]. Education and training must address the theory and application of SDM in district nursing, with distinctions made for older adults with and without reduced decision-making capacity [20].

Third, to create more efficient and productive consultations, in the education of district nurses effective communication skills should be central [50].

Fourth, to create goal-based and efficient intakes and evaluations, older adults and informal caregivers need preparation to prioritize their (independence) care goals in advance. The feasibility of existing preparatory tools [4] could be explored for district nursing care.

Fifth, developing a holistic geriatric assessment for district nursing is recommended to support the conduction of independence-related care needs and goals of older adults with MCC. The assessment should address both problems and capabilities of older adults and informal caregivers, and consider how the home and living environment can support aging in place.

### Strengths and limitations

A strength of this study is the versatility of analysis. We supplemented the qualitative analysis of verbal communication with quantitative analysis of non-verbal expressions, and comments from district nurses on the summarized results. Therefore, we could outline a broad picture. Another strength is the uniqueness of observations of person-centered SDM between district nurses and older adults on interventions to optimize independence. A weakness might be that we only have 16 district nurses with older adults on camera. A larger group may be needed to provide more robust findings. Given the low number of people who wanted to be filmed, another shortcoming may be that the district nurses who were permitted to be filmed had the most experience with SDM regarding the topic under study. This may have influenced study results.

## Conclusion

District nurses in this study engaged in shared decision-making (SDM) with a friendly approach, but discussions about independence were unstructured and often driven by older adults or referrers. A person-centered SDM model is needed to guide decisions supporting independence in older adults with MCC. This model should include preparatory and introductory steps and be co-developed with district nurses and older adults. Building trust, identified as fundamental by nurses, must be central to the model and reinforced through education and training. Additionally, implementing a holistic geriatric assessment tailored to district nursing and enhancing communication skills are recommended to better address the complex needs of this population.

## Supplementary Information

Below is the link to the electronic supplementary material.

Supplementary Material 1: Additional File 1. Questionnaire to collect characteristics of participants and description of the care need according to referrer, patient, informal caregiver and district nurse

Supplementary Material 2: Additional File 2. The observation scheme for verbal communication in the district nurse-older adult/informal caregiver communication during SDM

Supplementary Material 3: Additional File 3. The observation scheme for non-verbal behavior in the district nurse-older adult/informal caregiver communication

Supplementary Material 4: Additional File 4. Final Code Tree with deductive codes, themes, sub-themes and summary of the results

Supplementary Material 5: Additional File 5. Waived Approval of the study

## Data Availability

The data that support the findings of this study are available from the corresponding author upon reasonable request.

## Abbreviations

ADL: Activities of Daily Living
CM: Case Management
CN: Care Need
COPD: Chronic Obstructive Pulmonary Disease
DN: District Nurse
GP: General Practitioner
GR: Geriatric Rehabilitation
IC: Informal Caregiver
Pt: Patient
SDM: Shared Decision-Making
