# Supplementary material for: Person-centered shared decision-making in district nursing care on interventions to support independence in older adults with multiple chronic conditions: a video observation study
Source: BMC Nurs. 2025 Sep 26;24:1189. doi: 10.1186/s12912-025-03778-3 (PMC12465545; doi:10.1186/s12912-025-03778-3)
Supplement: Supplementary file 1 — Supplementary Material 1: Additional File 1. Questionnaire to collect characteristics of participants and description of the care need according to referrer, patient, informal caregiver and district nurse [file 12912_2025_3778_MOESM1_ESM.docx]

Additional file 1. Questionnaire to collect characteristics of participants and description of the care need according to referrer, patient, informal caregiver and district nurse

**Questionnaire older adults:**

How old are you?

What is your gender?

What is your marital status?

Do you have an informal caregiver?

What are the tasks of the informal caregiver (if the informal caregiver was present, this was discussed with the informal caregiver too)?

What care do you need, according to you?

How many times a week do you receive district nursing care?

**Questionnaire informal caregivers who attended during the observation:**

What is your relation to the patient?

What informal care tasks do you perform?

What care does the patient need, according to you?

**Questionnaire district nurse:**

How old are you?

What is your gender?

What care does the patient need, according to you?

What care does the patient need, according to the referrer?

For how many years are you working in district nursing care?
