# Supplementary material for: Person-centered shared decision-making in district nursing care on interventions to support independence in older adults with multiple chronic conditions: a video observation study
Source: BMC Nurs. 2025 Sep 26;24:1189. doi: 10.1186/s12912-025-03778-3 (PMC12465545; doi:10.1186/s12912-025-03778-3)
Supplement: Supplementary file 2 — Supplementary Material 2: Additional File 2. The observation scheme for verbal communication in the district nurse-older adult/informal caregiver communication during SDM [file 12912_2025_3778_MOESM2_ESM.docx]

Additional file 2. The observation scheme for verbal communication in the district nurse-older adult/informal caregiver communication during SDM

| **1 Preparation** **prior to the conversation**  Patient formulates personal goals, and related health and care goals  Healthcare professional identifies history of care and care problems |
| --- |
| **2 Team talk**  Healthcare professional introduces the decision-making process  Healthcare professional identifies the discussion partner |
| **3 Goal talk**  Healthcare professional identifies patient values and fundamental goals  Healthcare professional identifies the capacities of the patient  Healthcare professional identifies care problems, and related care goals  Healthcare professional identifies the prioritization of care goals |
| **4 Option talk**  Healthcare professional summarises and offers choices to address the care goals  Healthcare professional discusses personalized care options and patient participation |
| **5 Decision talk**  Healthcare professional connects to patient's values and goals in care  Healthcare professional and patient make shared decisions on nursing interventions |
| **6 Evaluation talk**  Healthcare professional initiates an evaluation of the decision and SDM process |
