# Supplementary material for: Person-centered shared decision-making in district nursing care on interventions to support independence in older adults with multiple chronic conditions: a video observation study
Source: BMC Nurs. 2025 Sep 26;24:1189. doi: 10.1186/s12912-025-03778-3 (PMC12465545; doi:10.1186/s12912-025-03778-3)
Supplement: Supplementary file 3 — Supplementary Material 3: Additional File 3. The observation scheme for non-verbal behavior in the district nurse-older adult/informal caregiver communication [file 12912_2025_3778_MOESM3_ESM.docx]

Additional file 3. The observation scheme for non-verbal behavior in the district nurse-older adult/informal caregiver communication

| **Code** | **Meaning** | **Description** |
| --- | --- | --- |
| 1. Making eye contact | Interest | Start: looking at the older adult/informal caregiver  End: looking away from the older adult |
| 2. Nodding and shaking head affirmatively | Kindness  Interest  Encouragement to speak further | Start: nodding and shaking the head affirmatively in response to what older adult is telling  End: keeping the head still |
| 3. Smiling | Forge a good relationship | Start: corners of mouth up  End: mouth corners not up |
| 4. Forward leaning | Listening  Involvement | Start: upper body toward older adult or informal caregiver  End: upright sitting position |
| 5. Affective touch | Affection  Care | Start: a friendly touch not related to caregiving  End: not touching |
| 6. Instrumental touch | Touch to perform a nursing task | Start: an occupational touch End: not touching |
| 7. Explaining with gestures | Supporting verbatim explanations | Start: making hand gestures to support spoken words  End: hands steady |
