# Supplementary material for: Person-centered shared decision-making in district nursing care on interventions to support independence in older adults with multiple chronic conditions: a video observation study
Source: BMC Nurs. 2025 Sep 26;24:1189. doi: 10.1186/s12912-025-03778-3 (PMC12465545; doi:10.1186/s12912-025-03778-3)
Supplement: Supplementary file 4 — Supplementary Material 4: Additional File 4. Final Code Tree with deductive codes, themes, sub-themes and summary of the results [file 12912_2025_3778_MOESM4_ESM.docx]

Additional file 4. Code tree

| Deductive codes | Themes | Sub-themes | Summary of observations |
| --- | --- | --- | --- |
|  |  |  |  |
| Preparation prior to the conversation | Being informed at start of the conversation |  | Older adults are unprepared. Most of them have never thought about their independence (care) needs. They mention not to know what district nursing care can offer/do. District nurses are well informed about the medical history and care request of the referrer. |
| Team talk | Initiating the conversation | Initiating the conversation | There is no talk of decisions being made. |
|  |  |  | District nurses often state that the care plan is about the client and their wishes are central. How to indicate wishes and care needs and why is not told. |
|  |  | Structuring the conversation | Often the patient sets the structure and the patient talks incessantly. |
|  |  |  | Structure of conversation and manner of decision making is not discussed. |
|  |  | Introducing information sources and discussion topics | No talk about independent functioning as a central topic for district nursing care. No introduction of SDM about interventions to support independence. |
|  |  |  | Introduction of what district nursing can do does not happen. Therefore, what can be discussed is not told. |
|  |  |  | Topics of conversation determined by district nurse: reporting, transfer, medical information, PREM. |
|  |  |  | Especially a physical focus of the conversation: the referrer's care request. |
|  | Exploring care needs |  | Patient did not think about personal goals and care goals in advance |
|  |  |  | Patient finds it difficult to indicate what district nursing can do/ what to expect from district nursing care. |
|  | Demonstrating attentiveness to the older adult | Exploring what matters to older adults | The district nurse keeps the atmosphere friendly and remains approachable: lots of laughter, smiling, encouraging words and room to keep talking. |
|  |  |  | Laughing along with the client, e.g. asking about the interior of the house, complimenting the garden. Lots of jokes and empathetic comments. |
|  |  |  | In general: lots of attention and time. No rush. |
|  |  |  | The perception of illness is asked a lot by district nurses. |
|  |  | Having conversations at the patient's level | District nurse usually tries to talk at the client's level, but sometimes uses unclear terms. |
| Goal talk | Identifying care problems and care goals |  | Client is given much opportunity to talk about illness experiences, problems. This digression is not channeled. All problems seem to matter equally. The many problems are not prioritized with clients. Clients ventilate their hearts but often nothing is done about it. |
|  |  |  | Independence (care) goals are not set. |
|  |  |  | No goals are filtered from the client's long stories. Many independence problems are mentioned. However, district nurses let client talk. There is no questioning from the district nurses about care goal/care demand. |
