# Supplementary material for: Person-centered shared decision-making in district nursing care on interventions to support independence in older adults with multiple chronic conditions: a video observation study
Source: BMC Nurs. 2025 Sep 26;24:1189. doi: 10.1186/s12912-025-03778-3 (PMC12465545; doi:10.1186/s12912-025-03778-3)
Supplement: Supplementary file 5 — Supplementary Material 5: Additional File 5. Waived Approval of the study [file 12912_2025_3778_MOESM5_ESM.docx]

Additional file 5. Waived approval

**Person-centered shared decision-making in district nursing care on interventions to support independence in older adults with multiple chronic conditions: A video observation study**

To whom it may concern,

On behalf of the non-WMO Committee of the Medical Ethics Review Committee of Amsterdam University Medical Centers we are pleased to confirm that the Medical Research Involving Human Subjects Act (WMO) does not apply to the above mentioned study. This committee is supervised by the Medical Ethics Review committee and authorised by the board of directors of Amsterdam UMC.

The Medical Ethics Review Committee of Amsterdam University Medical Centers is registered with the US Office for Human Research Protections (OHRP) as IRB00013752. The FWA number assigned to Amsterdam UMC is FWA00032965.

Kind regards,

On behalf of the Non-WMO Research Committee Amsterdam UMC,

Prof. Dr. J.A.M. van der Post, chairman
